# Cornelian Cherry (*Cornus mas*) Fruit Extract Administration in Sleep Deprived Wistar Rats—Friend or Foe?

**DOI:** 10.3390/biology14101341

**Published:** 2025-10-01

**Authors:** Vlad Sever Neculicioiu, Irina Camelia Chiș, Ioana Alina Colosi, Alexandra Sevastre-Berghian, Luminita David, Mara Muntean, Ana-Maria Vlase, Remus Moldovan, Roxana Maria Decea, Carmen Costache, Horațiu Alexandru Colosi, Dan Alexandru Toc, Şoimiţa Mihaela Suciu, Simona Clichici

**Affiliations:** 1 Department of Microbiology, “Iuliu Hatieganu” University of Medicine and Pharmacy, 400349 Cluj-Napoca, Romania; 2 Department of Physiology, “Iuliu Hatieganu” University of Medicine and Pharmacy, 400006 Cluj-Napoca, Romania; 3 Research Centre for Advanced Chemical Analysis, Instrumentation and Chemometrics, Faculty of Chemistry and Chemical Engineering, Babes-Bolyai University, 400028 Cluj-Napoca, Romania; 4 Department of Cell and Molecular Biology, Faculty of Medicine, “Iuliu Hațieganu” University of Medicine and Pharmacy, 400349 Cluj-Napoca, Romania; 5 Department of Pharmaceutical Botany, Faculty of Pharmacy, “Iuliu Hațieganu” University of Medicine and Pharmacy, 8 Victor Babeș Street, 400012 Cluj-Napoca, Romania; 6 Division of Medical Informatics and Biostatistics, Department of Medical Education, “Iuliu Hațieganu” University of Medicine and Pharmacy, 400349 Cluj-Napoca, Romania

**Keywords:** REM sleep, PSD, plant preparations, antioxidants, Malondialdehyde, electron microscopy, Interleukin-6, Interleukin-1beta, Interleukin-4

## Abstract

**Simple Summary:**

While the negative effects of sleep deprivation on the brain are well known, the impact on other organs has been less studied. The use of dietary supplements has become increasingly common, even though scientific evidence for their overall health benefits is often limited. Antioxidant supplements may also have side effects and may sometimes act in unexpected ways depending on the biological environment or dose. In this study, we examined the effects of a Cornelian cherry (*Cornus mas*) fruit extract on several peripheral organs in rats exposed to prolonged REM sleep deprivation. After seven days of sleep loss, the animals showed signs of damage in most examined organs. This included oxidative stress and ultrastructural injury in the liver, oxidative stress in the kidneys, spleen, and aorta, as well as increased levels of inflammatory cytokines in different organs. Treatment with *Cornus mas* partially protected the liver but had limited effects elsewhere. In the aorta, the extract partly restored some antioxidant parameters but also unexpectedly increased lipid oxidative damage. These findings highlight that the effects of sleep deprivation extend beyond the brain. Furthermore, while based on limited data, extracts with antioxidant properties may prove to be a double-edged sword in some cases.

**Abstract:**

Supplement use has increased in recent years, despite limited evidence for its broad health benefits. Furthermore, exogenous antioxidants may determine pro-oxidant effects, depending on various factors such as dose, circadian window, and presence of metal ions. Although the effects of sleep deprivation (SD) on the brain are well-documented, its impact on peripheral organs remains relatively underexplored. The goal of this study was to evaluate the effects of a *Cornus mas (C. mas)* fruit extract on multiple peripheral sites in rats undergoing paradoxical sleep deprivation (PSD). Male Wistar rats were randomly distributed in four groups, including control, *C. mas* (CM), sleep deprivation (SD), and sleep deprivation and *C. mas* (SD+CM) (n = 7/group). Seven days of PSD were associated with ultrastructural liver injury and evidence of oxidative dysfunction in several organs: liver, kidney, spleen, and aorta. These alterations were accompanied by marked increases in the evaluated cytokines, including testicular Interleukin-1β, hepatic Interleukin-6, and aortic Interleukin-4. Although the *C. mas* extract largely maintained hepatic ultrastructure, its effects on other organs were limited. In the aorta, it normalized GSSG values but was also associated with a significant increase in lipid peroxidation. These findings highlight both the systemic impact of SD and caution against assuming uniform benefits of exogenous antioxidants across organ systems in this context.

## 1. Introduction

In recent years, sleep deprivation (SD) has been recognised as an epidemic by the Centers for Disease Control and Prevention (CDC), thus highlighting its role in a wide range of adverse health effects such as hypertension, obesity, and cardiovascular diseases as well as an overall increased mortality [1]. In accordance with this, both previous and recent epidemiological trends suggest that more than one-third of adults have experienced an increased, yet relatively stable, prevalence of short sleep over the past decade [2,3]. Additionally, REM SD has been identified as a silent and underrecognized epidemic, driven by various factors including substance abuse, various medications, sleep disorders, and lifestyle factors [4].

Oxidative stress (defined as an imbalance between oxidants and antioxidants) has been clearly implicated in the onset and progression of a wide range of chronic conditions, extending from cardiovascular to neurodegenerative diseases and cancer (reviewed in [5]). However, reactive oxygen species (ROS) play vital roles at physiological concentrations, ranging from redox signalling and cellular homeostasis to roles in the immune system, aging, CNS signalling, and fertility (reviewed in [6,7]).

The expression “sleep is of the brain, by the brain and for the brain” [8] encompassed the previously held paradigm regarding the importance of sleep for the central nervous system (CNS). However, recent data from rodent models seem to suggest that the effects of SD extend beyond the CNS, also encompassing peripheral sites, particularly in relation to oxidative stress [9,10]. SD or sleep restriction has been shown to determine an oxidative burden in a wide range of rodent peripheral sites such as liver, aorta, submandibular glands, and others, with Wistar rats being one of the most frequently employed models [9]. Additionally, while relatively limited, these effects seem translational in human models in both the endothelium [11] and serum [12,13]. While various exogenous antioxidants from natural sources have shown promise in modulating the effects of SD in rodent models (e.g., Tualang honey, *Withania somnifera*, *Camellia sinensis,* etc) [14,15,16], there is a paucity of clinical trials examining these effects in humans.

It is well established that a balanced diet offers numerous health benefits, with both quantity and variety of fruits and vegetables being essential for reducing the risk of various chronic health conditions and overall mortality [17,18]. Furthermore, both dietary intake and blood concentrations of different antioxidants (from fruit and vegetable consumption) show a similar health-promoting effect [19]. Consequently, dietary supplement use has increased substantially in the past decades, with over half of US adults taking a supplement [20]. Specifically, antioxidant use is also highly prevalent in specific populations such as performance athletes [21] or cancer patients [22]. However, the evidence regarding the use of high doses of exogenous antioxidants in clinical trials is inconsistent for most health conditions. In some cases, antioxidants may even be harmful, potentially acting as pro-oxidants [23], decreasing the health benefits of exercise [21], or interfering with cancer treatments [22]. The cytoprotective effects of exogenous antioxidants appear to be better explained by their pro-oxidant actions and the subsequent hermetic activation of cellular defence pathways, such as Nrf2-ARE, rather than by their direct antioxidant effects. However, at high doses, the pro-oxidant activity might surpass the adaptive capacity of cells and cause cytotoxicity [24]. Globally, antioxidants may provide pro-oxidant effects depending on multiple factors such as concentration, redox potential, presence of other antioxidants and transition metals, and the activity of endogenous antioxidant systems, ultimately leading to “antioxidant-induced stress” [25]. Furthermore, chronic stimulation and activation of endogenous antioxidant pathways such as Nrf2 could lead to “reductive stress” [21].

This study was part of a larger proof-of-concept project aimed at investigating the effects of a *Cornus mas* fruit extract as a viable modulator in a rodent model of REM sleep deprivation. We previously reported relatively modest effects of this extract mainly on behavioural alterations (hyperlocomotion, hyperactivity) and brain histological and ultrastructural changes following seven days of PSD [26]. This study aimed to further investigate additional outcomes across multiple peripheral organs, specifically the liver, aorta, kidney, spleen, and testicle, while also assessing the potential modulatory effects of the administered extract on redox balance and inflammatory responses within these tissues.

## 2. Materials and Methods

### 2.1. Cornus Mas Fruit Extract–Preparation, Characterisation and In Vitro Antioxidant Activity

The fruit extract was prepared from fresh cornelian cherry fruits (*Cornus mas*) (Identified by Assistant Professor Ana-Maria Vlase, PhD—Herbarium code UMF Cluj-Napoca 66.1.1.1/06.2023) as previously described [26]. Briefly, the paste obtained from destoned fruits was mixed with acetone (ratio 1:2.5), stirred in a magnetic stirrer for four hours at room temperature, filtered, and subsequently vacuum concentrated until acetone was completely eliminated at 40 °C. Total polyphenol content of the obtained extract was determined through the Folin-Ciocalteu method in triplicate, as previously described [26], revealing a value of 2.55 ± 0.066 g gallic acid equivalents (GAE)/100 mL of extract.

The phytoconstituent profile of the extract was further analyzed using multiple methodologies on liquid chromatography-tandem mass spectrometry (LC-MS/MS). The analysis was performed on an Agilent Technologies 1100 HPLC Series system (Agilent, Santa Clara, CA, USA) coupled with an Agilent Ion Trap 1100 SL mass spectrometer (LC/MSD Ion Trap VL), as previously described. All analyses were performed in triplicate. The analysis revealed the presence of 14 phenolic compounds, with the major identified compounds being caffeic acid (70.998 ± 7.80 µg/mL), chlorogenic acid (70.647 ± 9.18 µg/mL), gallic acid (49.49 ± 6.43 µg/mL), isoquercitrin (37.476 ± 1.12 µg/mL), and caftaric acid (35.992 ± 0.71 µg/mL). Among anthocyanins, only cyanidin-3-glucoside was identified in high amounts (1162.241 ± 34.86 µg/mL) [26].

The in vitro antioxidant capacity of the extract was further determined through the DPPH (2,2-diphenyl-1-picrylhydrazyl) assay in triplicate, as previously outlined. The cherry extract exhibited an in vitro antioxidant activity of 4.8497 ± 0.0094 mg Trolox equivalent (TE)/mL of extract [26].

### 2.2. Experimental Animals and Study Design

All experimental procedures were approved by the Animal Ethics Board of “Iuliu Hatieganu” University of Medicine and Pharmacy (Authorization No 370/13.06.2023). All animal work complied with the EU Directive 2010/63 on the protection of animals used for scientific purposes and the local applicable legislative framework and respected the three Rs of ethical animal research.

Experimental animals were acquired from the animal facility of the University of Medicine and Pharmacy “Iuliu Hatieganu”, Cluj-Napoca, Romania. Twenty-eight Wistar adult male rats (200–250 g) were group housed under standard laboratory conditions (room temperature 24 ± 2 °C and 12 h light—12 h dark cycles), with free access to standard normocaloric pellet diet and drinking water for the duration of the experiment.

**Figure 1:**
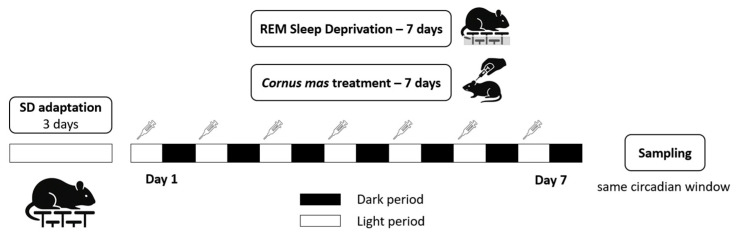
Experimental timeline. The study included four groups (n = 7): Control, *Cornus mas* (CM), Sleep deprivation (SD), Sleep deprivation and *Cornus mas* (SD+CM). The SD and SD+CM groups were exposed to REM SD for seven continuous days through the modified multiple small platform paradigm (MMSP). Symbols indicate *C. mas* treatment, which was administered as one daily dose of extract via oral gavage (1.2 mL/kg bw/day) at the start of the light phase. *C. mas* treatment was given to the CM and SD+CM groups, while the Control and SD groups received daily sham treatment with asimilar dose of distilled water.

Upon arrival, animals were acclimated for seven days before the start of the experimental procedures. Animals were randomised (GraphPad QuickCalcs randomiser—https://www.graphpad.com/quickcalcs/randomize1/ accessed on 30 November 2023) into four groups: Control, *Cornus mas* (CM), Sleep deprivation (SD), Sleep deprivation with *Cornus mas* (SD+CM). Each experimental group contained seven animals. SD and SD+CM groups were sleep deprived through the Modified Multiple Small Platforms (MMSP) protocol for seven continuous days and the CM and SD+CM were treated with *Cornus mas* fruit extract for seven days (1.2 mL/kg bw/day; ~30 mg polyphenols/kg bw/day). The control and SD groups were sham-treated with a similar dose of distilled water through oral gavage, in order to minimize potential confounding effects of intervention-induced stress. This procedure was conducted in the same manner as the *C. mas* extract administration. The detailed experimental timeline is presented in Figure 1. The same researchers performed all experimental procedures during the light phase. A single animal was designated as the experimental unit.

The study protocol was designed a priori, not registered, and had as inclusion criteria healthy experimentally naïve male rodents of comparable age and body weight. Treatment order and sample collection were randomised. Outcome measures were evaluated in several peripheral body sites (liver, kidney, spleen, aorta, testicle): oxidative stress parameters, pro- and anti-inflammatory cytokines. Ultrastructural changes were evaluated in the liver.

### 2.3. Induction of Sleep Deprivation

**Figure 2:**
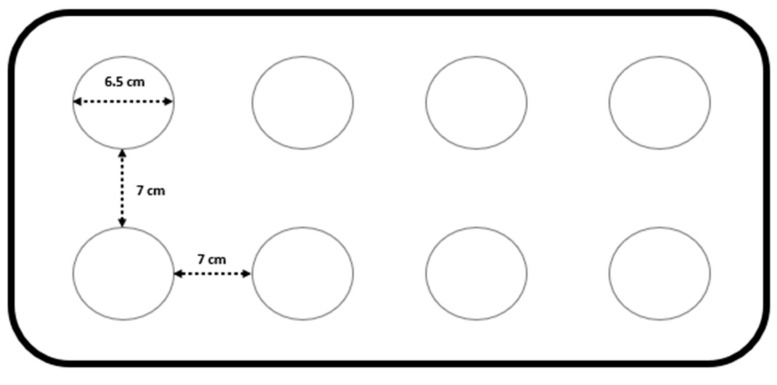
Schematic representation of the paradoxical sleep deprivation tanks.

Paradoxical sleep deprivation was performed through the Modified Multiple Small Platforms (MMSP) method [27] for seven continuous days, as previously described [26]. Briefly, groups of 3–4 socially stable animals were placed on 6.5 cm diameter platforms surrounded by water that inhibited REM sleep due to muscle atonia. Animals were acclimated for 2 h daily during the light phase for three days prior to the start of the experimental sleep deprivation, in order for the animals to learn to balance on the platforms and to reduce unnecessary falls in the water and reduce potential confounding effects associated with exposure to a novel environment [28]. The water in the tanks was changed daily and the tanks were thoroughly disinfected with 70% alcohol each day. A schematic representation of the SD tanks is available in Figure 2.

### 2.4. Sample Collection

At the end of the SD paradigm, all animals were euthanized in the same circadian window (8–12 AM) via intraperitoneal administration of anaesthetics (ketamine 90 mg/kg bw and xylazine 10 mg/kg bw). Immediately following euthanasia, the peripheral organs (liver, kidney, spleen, aorta, testicle) were extracted on ice and further processed according to the intended determinations.

### 2.5. Transmission Electron Microscopy (TEM)

Liver samples were collected for qualitative TEM analysis (n = 3 in each group) and further processed as previously outlined [26]. Following fixation in 2.7% glutaraldehyde (0.1M phosphate buffer), samples were washed four times in the same buffer. After postfixation with 1.5% osmium tetroxide in 0.15 M phosphate buffer, the liver samples were dehydrated in graded acetone (30–100%) and embedded in EMbed 812 epoxy resin (Electron Microscopy Sciences, Hatfield, PA, USA). Ultrathin sections of 70–80 nm were obtained (Diatome A382 diamond knife—Diatome, Hatfield, PA, USA; Bromma 8800 ULTRATOME III—LKB, Stockholm, Sweden), mounted on 300-mesh copper grids, double-stained with uranyl acetate and lead citrate, examined at 80 kV using a JEOL JEM-100CX II transmission electron microscope (JEOL, Tokyo, Japan), and images were obtained with a MegaView G3 camera and Radius 2.1 software (Emsis, Münster, Germany).

### 2.6. Oxidative Stress Parameters

Dissected liver, kidney, spleen, aorta, and testicle samples were processed for the determination of several oxidative stress parameters: reduced Glutathione (GSH), oxidised Glutathione (GSSG), GSH/GSSG ratio, Catalase activity (CAT) and Malondialdehyde (MDA) (n = 7/group).

Immediately after sampling, tissues were homogenised on ice (POLYTRON PT 1200 E homogeniser—Kinematica, Malters, Switzerland; 50 mM TRIS and 10 mM EDTA buffer at pH 7.5), centrifuged at 1000 g, 10 min, 4 °C, and the resulting supernate was immediately stored in pre-labelled Eppendorf tubes at −80 °C for subsequent analysis. Supernate protein content was further determined as previously described [26,29].

MDA levels were measured fluorometrically (Perkin Elmer, Shelton, CT, USA) as a byproduct of lipid peroxidation, using thiobarbituric acid, as previously described [26,30]. Following one hour incubation in a boiling water bath in a solution of thiobarbituric acid and K_2_HPO_4_ buffer (10 mM 2-thiobarbituric acid and 75 mM K_2_HPO_4_ buffer at pH 3.0) and extraction with n-butanol, MDA values were determined fluorometrically (excitation 543 nm, Δλ 14 nm between excitation-emission) and expressed as nmol/mg protein.

The ratio between GSH and GSSG was determined as an extensively used marker of cellular health and cellular redox potential [31]. GSH and GSSG values were determined as previously outlined [26]. Fluorescence was measured (excitation—350 nm, emission—420 nm), GSH and GSSG values were determined with the use of a standard curve and expressed as nmol/mg protein.

CAT activity in the liver, kidney, spleen, aorta, and testicle samples was determined as previously outlined [32]. CAT activity was determined by monitoring the change in absorbance of a 10 mM H_2_O_2_ solution in 0.05 M potassium phosphate buffer (pH 7.4) at 240 nm. One unit of activity was defined as the amount of enzyme that decreases the absorbance by 0.43 at 25 °C over 3 min. The enzyme activity was expressed as U/g of protein using the following formula: CAT = A_240_/0.43 × 0.02.

### 2.7. Pro- and Anti-Inflammatory Cytokine Determination

Cytokines with mainly inflammatory (Interleukin-6, Interleukin-1β) and anti-inflammatory roles (Interleukin-4) were determined through rat-specific ELISA kits, as per the manufacturer’s instructions (n = 5/group): IL-6, IL-1β, IL-4 (E-EL-R0015, E-EL-R0012, E-EL-R0014; Elabscience, Houston, TX, USA). Absorbance readings were performed at 450 nm. Results were expressed as pg/mg protein.

### 2.8. Statistical Analysis

Group randomisation was performed with the online utility provided by GraphPad (GraphPad QuickCalcs randomiser—https://www.graphpad.com/quickcalcs/randomize1/ accessed on 30 November 2023).

Statistical analysis was performed in a blinded manner in GraphPad Prism version 9.3.0 on a Microsoft Windows 11 23H3 machine (GraphPad Software, San Diego, CA, USA—https://www.graphpad.com/). Data normality was assessed using the Kolmogorov–Smirnov and Shapiro–Wilk tests. Group differences were analyzed through two-way ANOVA (Sleep, Treatment, Sleep x Treatment interaction), reported as (F, η^2^, *p* values) and followed by Tukey’s post hoc test with *p* values adjusted for multiple comparisons. Data were expressed as mean ± SEM unless otherwise specified. Results were considered statistically significant at *p* < 0.05.

## 3. Results

### 3.1. Liver Ultrastructural Analysis

**Figure 3:**
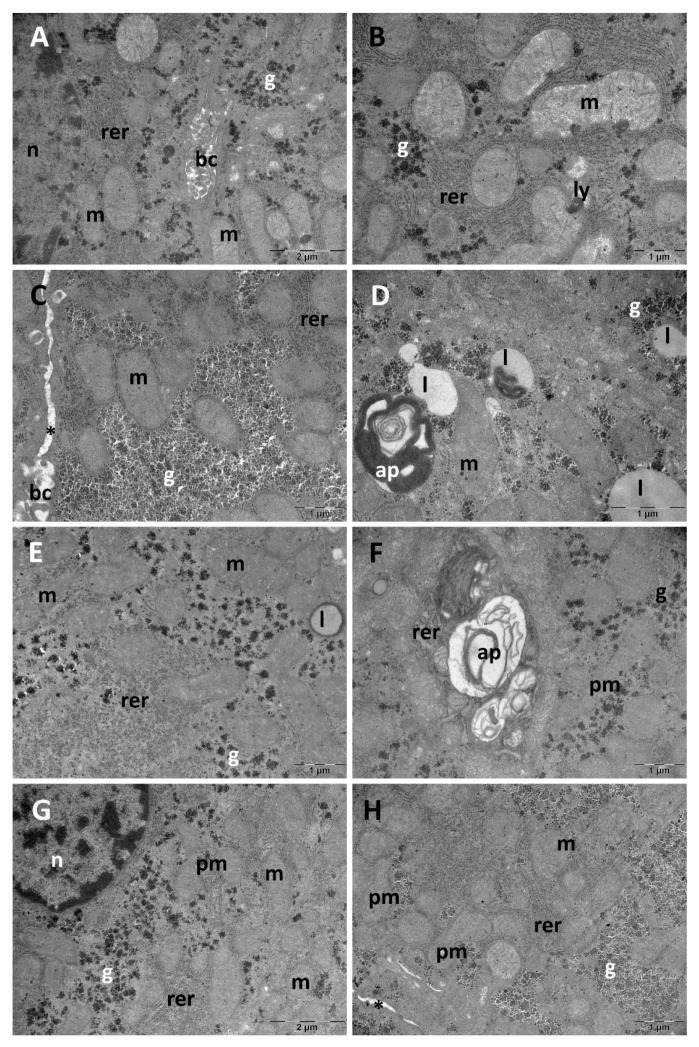
Ultrastructural investigation of the liver. Control (**A**,**B**); SD (**C**,**D**); CM (**E**,**F**); SD+CM (**G**,**H**); Control—Control group; CM—*Cornus mas* group; SD—Sleep deprivation group; SD+CM—Sleep deprivation with *Cornus mas* group; ap—autophagosome; bc—bile canaliculus; g—glycogen granules; l—lipid droplet; ly—primary lysosome; m—mitochondrion; n—nucleus; pm—polymorphic mitochondrion; rer—rough endoplasmic reticulum; *—wider intercellular space; Scale bars of 1–2 µm are presented in each individual panel.

Qualitative ultrastructural changes were evaluated in the liver (Figure 3). Normal hepatic ultrastructure was evident in the Control group with large, rounded nuclei and abundant cytoplasm in hepatocytes, round- or oval-shaped mitochondria with numerous small cristae. Additionally, rough endoplasmic reticulum profiles and glycogen granules were also noted, alongside occasional lipid droplets. Between adjacent hepatocytes, bile canaliculi were observed (Figure 3A,B). The most prominent ultrastructural alterations were observed in the SD group. In some areas of the tissue, glycogen accumulations were noted (Figure 3C). The lipid droplets were numerous and some were heterogeneous. Autophagosomes were also found and mitochondria were polymorphic. A wider intercellular space was noted between the hepatocytes in this group, most likely due to disorganization of some desmosomes (belt and spot) (Figure 3D). The CM group presented lipid droplets with an electron-dense outer rim (Figure 3E). Furthermore, occasional autophagosomes were noted, alongside polymorphic mitochondria (Figure 3F). In the SD+CM group, TEM analysis revealed an almost normal ultrastructural aspect. Some of the mitochondria were polymorphic, and glycogen accumulations were also present in some areas of the tissue. Additionally, a wider intercellular space was noted (Figure 3G,H).

### 3.2. Oxidative Stress Parameters and Cytokine Changes

**Figure 4:**
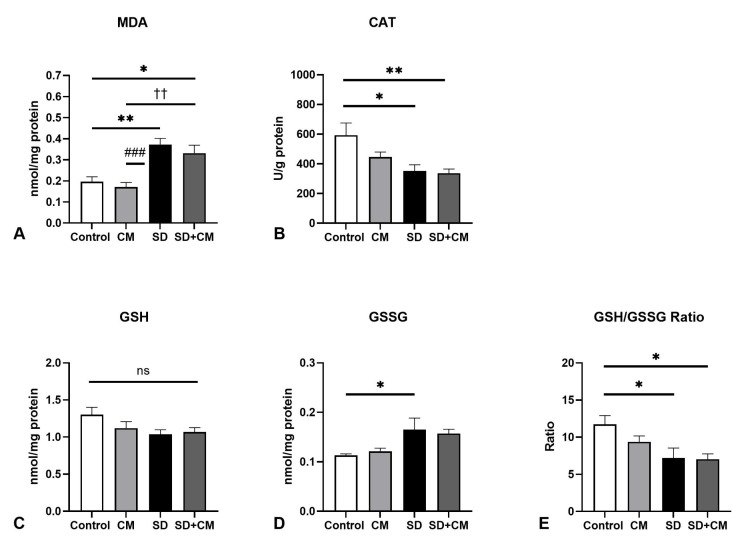
Liver redox balance parameters: (**A**) Malondialdehyde (MDA), (**B**) Catalase (CAT), (**C**) Reduced Glutathione (GSH), (**D**) Oxidised Glutathione (GSSG), (**E**) GSH/GSSG ratio; Control—Control group; CM—*Cornus mas* group; SD—Sleep deprivation group; SD+CM—Sleep deprivation with *Cornus mas* group; Results are expressed as mean ± SEM (n = 7/group); two-way ANOVA followed by Tukey’s post-test; *p*-values adjusted for multiple comparisons; * *p* < 0.05, ** *p* < 0.01 vs. Control; ^###^ *p* < 0.001 vs. SD; ^††^ *p* < 0.01 vs. CM; ns = not significant.

**Figure 5:**
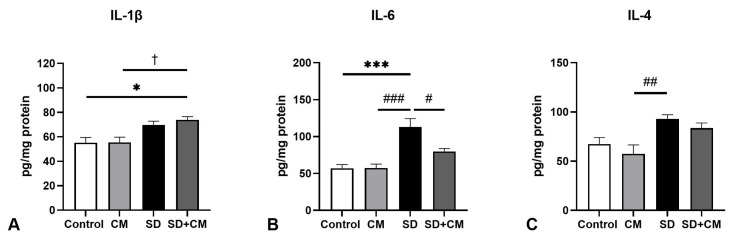
Liver anti- and pro-inflammatory cytokine levels: (**A**) Interleukin-1β (IL-1β), (**B**) Interleukin-6 (IL-6), (**C)** Interleukin-4 (IL-4); Control—Control group; CM—*Cornus mas* group; SD—Sleep deprivation group; SD+CM—Sleep deprivation with *Cornus mas* group; Results are expressed as mean ± SEM (n = 5/group); two-way ANOVA followed by Tukey’s post-test; *p*-values adjusted for multiple comparisons; * *p* < 0.05, *** *p* < 0.001 vs. Control; ^#^ *p* < 0.05, ^##^ *p* < 0.01, ^###^ *p* < 0.001 vs. SD; ^†^ *p* < 0.05 vs. CM.

**Figure 6:**
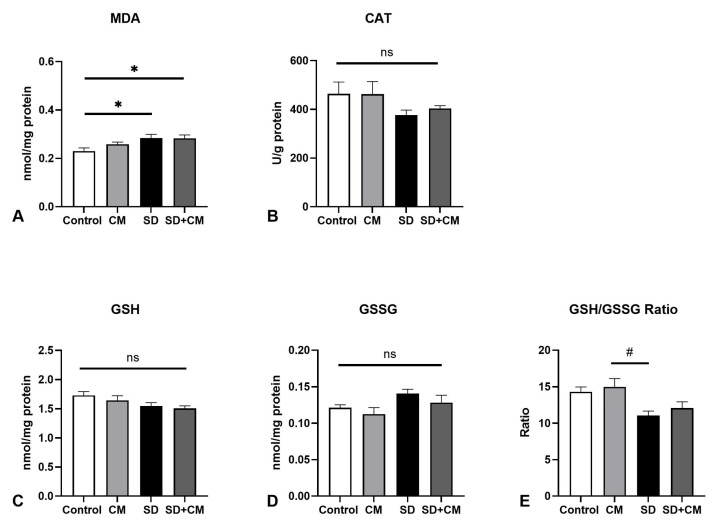
Kidney redox balance parameters: (**A**) Malondialdehyde (MDA), (**B**) Catalase (CAT), (**C**) Reduced Glutathione (GSH), (**D**) Oxidised Glutathione (GSSG), (**E**) GSH/GSSG ratio; Control—Control group; CM—*Cornus mas* group; SD—Sleep deprivation group; SD+CM—Sleep deprivation with *Cornus mas* group; Results are expressed as mean ± SEM (n = 7/group); two-way ANOVA followed by Tukey’s post-test; *p*-values adjusted for multiple comparisons; * *p* < 0.05 vs. Control; ^#^ *p* < 0.05 vs. SD; ns = not significant.

**Figure 7:**
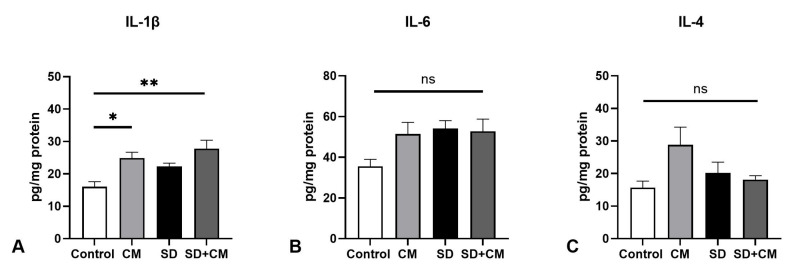
Kidney anti- and pro-inflammatory cytokine levels: (**A**) Interleukin-1β (IL-1β), (**B**) Interleukin-6 (IL-6), (**C**) Interleukin-4 (IL-4); Control—Control group; CM—*Cornus mas* group; SD—Sleep deprivation group; SD+CM—Sleep deprivation with *Cornus mas* group; Results are expressed as mean ± SEM (n = 5/group); two-way ANOVA followed by Tukey’s post-test; *p*-values adjusted for multiple comparisons; * *p* < 0.05, ** *p* < 0.01 vs. Control; ns = not significant.

**Figure 8:**
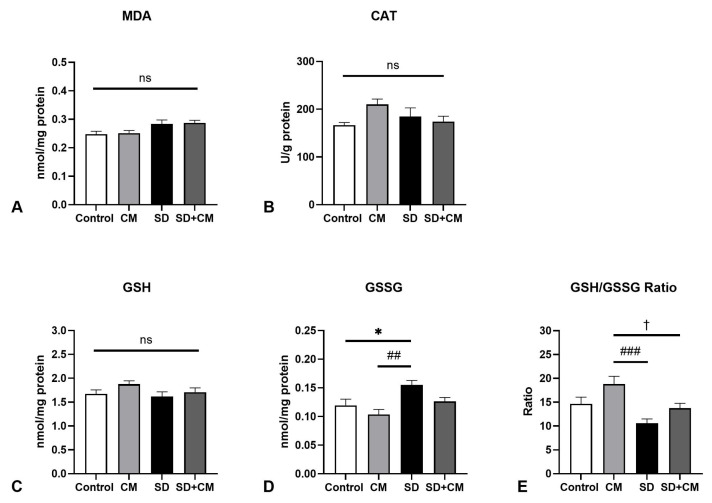
Spleen redox balance parameters: (**A**) Malondialdehyde (MDA), (**B**) Catalase (CAT), (**C**) Reduced Glutathione (GSH), (**D**) Oxidised Glutathione (GSSG), (**E**) GSH/GSSG ratio; Control—Control group; CM—*Cornus mas* group; SD—Sleep deprivation group; SD+CM—Sleep deprivation with *Cornus mas* group; Results are expressed as mean ± SEM (n = 7/group); two-way ANOVA followed by Tukey’s post-test; *p*-values adjusted for multiple comparisons; * *p* < 0.05 vs. Control; ^##^ *p* < 0.01, ^###^ *p* < 0.001 vs. SD; ^†^ *p* < 0.05 vs. CM; ns = not significant.

**Figure 9:**
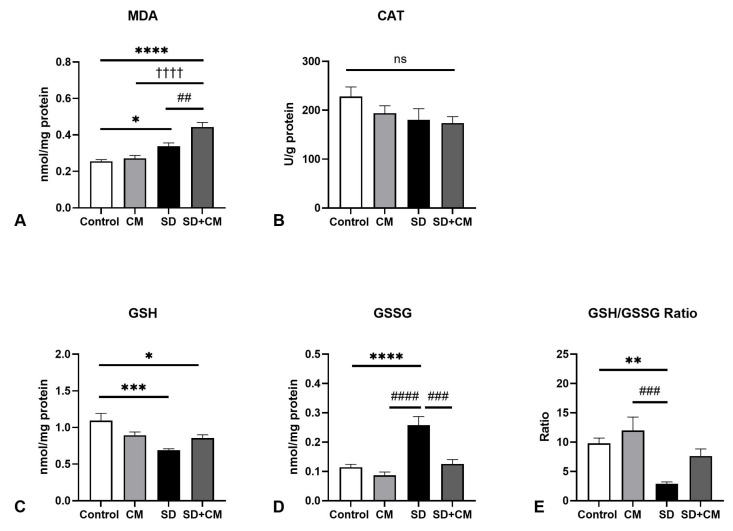
Aorta redox balance parameters: (**A**) Malondialdehyde (MDA), (**B**) Catalase (CAT), (**C**) Reduced Glutathione (GSH), (**D**) Oxidised Glutathione (GSSG), (**E**) GSH/GSSG ratio; Control—Control group; CM—*Cornus mas* group; SD—Sleep deprivation group; SD+CM—Sleep deprivation with *Cornus mas* group; Results are expressed as mean ± SEM (n = 7/group); two-way ANOVA followed by Tukey’s post-test; *p*-values adjusted for multiple comparisons; * *p* < 0.05, ** *p* <0.01, *** *p* < 0.001, **** *p* < 0.0001 vs. Control; ^##^ *p* < 0.01, ^###^ *p* < 0.001, ^####^ *p* < 0.0001 vs. SD; ^††††^ *p* < 0.0001 vs. CM; ns = not significant.

**Figure 10:**
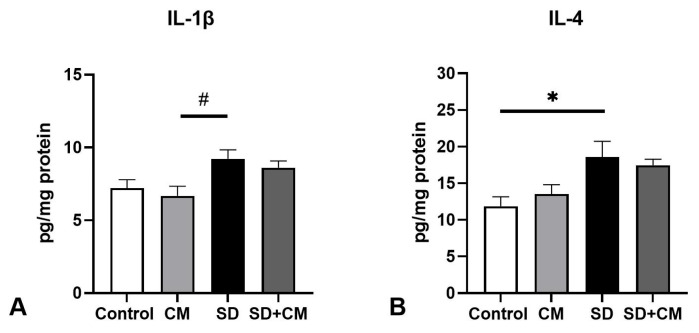
Aorta anti- and pro-inflammatory cytokine levels: (**A**) Interleukin-1β (IL-1β), (**B**) Interleukin-4 (IL-4); Control—Control group; CM—*Cornus mas* group; SD—Sleep deprivation group; SD+CM—Sleep deprivation with *Cornus mas* group; Results are expressed as mean ± SEM (n = 5/group); two-way ANOVA followed by Tukey’s post-test; *p*-values adjusted for multiple comparisons; * *p* < 0.05 vs. Control; ^#^ *p* < 0.05 vs. SD.

**Figure 11:**
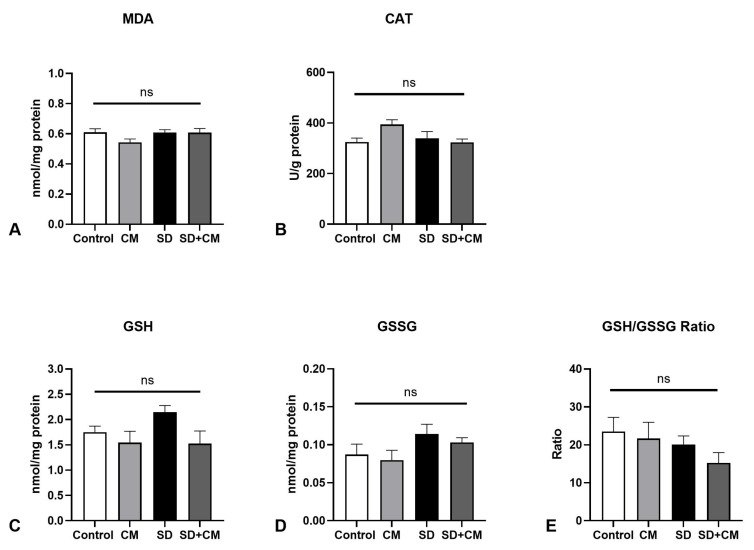
Testicle redox balance parameters: (**A**) Malondialdehyde (MDA), (**B**) Catalase (CAT), (**C**) Reduced Glutathione (GSH), (**D**) Oxidised Glutathione (GSSG), (**E**) GSH/GSSG ratio; Control—Control group; CM—*Cornus mas* group; SD—Sleep deprivation group; SD+CM—Sleep deprivation with *Cornus mas* group; Results are expressed as mean ± SEM (n = 7/group); two-way ANOVA followed by Tukey’s post-test; *p*-values adjusted for multiple comparisons; ns = not significant.

**Figure 12:**
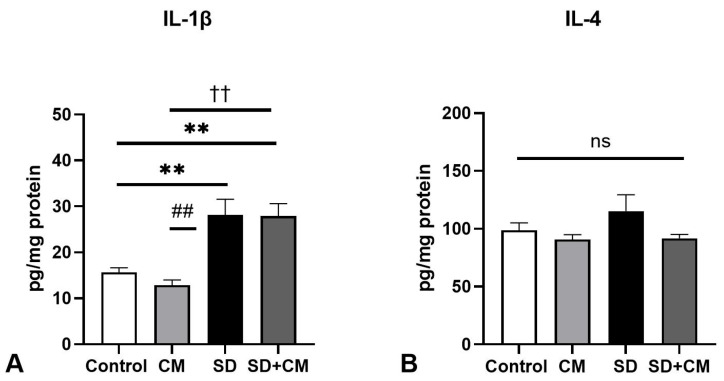
Testicle anti- and pro-inflammatory cytokine levels: (**A**) Interleukin-1β (IL-1β), (**B**) Interleukin-4 (IL-4); Control—Control group; CM—*Cornus mas* group; SD—Sleep deprivation group; SD+CM—Sleep deprivation with *Cornus mas* group; Results are expressed as mean ± SEM (n = 5/group); two-way ANOVA followed by Tukey’s post-test; *p*-values adjusted for multiple comparisons; ** *p* < 0.01 vs. Control; ^##^ *p* < 0.01 vs. SD; ^††^ *p* < 0.01 vs. CM; ns = not significant.

Multiple oxidative stress-related parameters (GSH, GSSG, GSH/GSSG ratio, CAT, MDA) were evaluated in the liver, kidney, spleen, aorta, and testicle. Cytokines with mainly pro- or anti-inflammatory roles (IL-1β, IL-6, IL-4) were evaluated in the liver, kidney, aorta, and testicle samples. Results are presented in Figure 4, Figure 5, Figure 6, Figure 7, Figure 8, Figure 9, Figure 10, Figure 11 and Figure 12.

In the case of liver redox parameters (Figure 4), two-way ANOVA revealed a significant main effect of sleep in regards to MDA (F = 35.35, η^2^ = 0.58, *p* < 0.0001), GSSG (F = 11.27, η^2^ = 0.31, *p* = 0.0026), GSH/GSSG ratio (F = 10.90, η^2^ = 0.29, *p* = 0.0030) and CAT (F = 11.71, η^2^ = 0.29, *p* = 0.0022). No significant effect of treatment or significant interaction effect was observed in the evaluated parameters. In comparison to control, SD resulted in a significant increase in MDA (*p* < 0.01) and GSSG values (*p* < 0.05), coupled with significant decreases in CAT (*p* < 0.05) and GSH/GSSG ratio (*p* < 0.05), suggesting an altered redox status in the liver. Furthermore, in comparison to the CM group, SD resulted in significantly increased MDA values (*p* < 0.001). Compared to SD alone, treatment with *C. mas* revealed a tendency towards normalisation in the case of MDA levels (*p* > 0.05).

With respect to liver cytokine levels (Figure 5), two-way ANOVA revealed a significant effect of sleep in regards to all three examined cytokines IL-1β (F = 19.80, η^2^ = 0.54, *p* = 0.0004), IL-6 (F = 31.66, η^2^ = 0.53, *p* < 0.0001) and IL-4 (F = 15.68, η^2^ = 0.46, *p* = 0.0011). Furthermore, IL-6 levels showed an additional significant main effect of treatment (F = 5.58, η^2^ = 0.09, *p* = 0.031), as well as a significant interaction effect between sleep and treatment (F = 5.78, η^2^ = 0.09, *p* = 0.02). Compared to control, SD determined increases in IL-1β (*p* > 0.05), IL-4 (*p* > 0.05), and IL-6 (*p* < 0.001), possibly pointing towards a proinflammatory state in the liver. In addition, SD determined a significant increase in both IL-6 (*p* < 0.001) and IL-4 (*p* < 0.01) in comparison to CM. Compared to SD alone, *C. mas* administration in the SD+CM group determined a normalisation of IL-6 values increased by SD (SD vs. SD+CM, *p* < 0.05 and SD+CM vs. Control, *p* > 0.05), pointing towards a potential anti-inflammatory effect of the extract.

In the case of kidney redox parameters (Figure 6), two-way ANOVA revealed a significant effect of sleep in the case of MDA (F = 8.61, η^2^ = 0.25, *p* = 0.0072), GSH (F = 6.34, η^2^ = 0.2, *p* = 0.018), GSSG (F = 5.53, η^2^ = 0.17, *p* = 0.02) and GSH/GSSG (F = 13.64, η^2^ = 0.35, *p* = 0.001). No significant effect of treatment or interaction effect was observed in the examined parameters. Compared to Control, SD determined an increase in MDA and GSSG, coupled with decreases in GSH, CAT, and GSH/GSSG ratio. However, statistical significance was reached only regarding MDA (*p* < 0.05), suggesting potential oxidative lipid damage in the kidneys. Furthermore, the GSH/GSSG ratio was significantly decreased by SD in comparison to the CM group (*p* < 0.05).

In the case of kidney cytokines (Figure 7), two-way ANOVA revealed a significant main effect of sleep and treatment in the case of IL-1β (F = 6.18, η^2^ = 0.16, *p* = 0.02 and F = 15.31, η^2^ = 0.4, *p* = 0.0012) and a significant interaction effect in the case of IL-4 (F = 5.04, η^2^ = 0.20, *p* = 0.039). In this tissue, IL-1β levels were significantly elevated in the CM group relative to controls (*p* < 0.05).

Regarding spleen redox balance (Figure 8), two-way ANOVA revealed a significant main effect of sleep in the case of MDA (F = 11.51, η^2^ = 0.32, *p* = 0.002). A significant main effect of treatment and sleep were further evidenced in regards to GSSG (F = 6.55, η^2^ = 0.15, *p* = 0.01 and F = 11.31, η^2^ = 0.26, *p* = 0.0026) and GSH/GSSG ratio (F = 8.15, η^2^ = 0.18, *p* = 0.008 and F = 12.72, η^2^ = 0.28, *p* = 0.0016). Furthermore, a significant interaction effect between sleep and treatment was outlined in the case of spleen CAT activity (F = 4.78, η^2^ = 0.15, *p* = 0.03). Similar to the other body sites, SD determined a significant increase in GSSG (*p* < 0.05) in comparison to the control group, possibly pointing towards redox imbalance. Furthermore, SD determined a significant increase in GSSG coupled with a significant decrease in GSH/GSSG ratio in comparison to the CM group (*p* < 0.01 and *p* < 0.001, respectively).

In regards to aorta redox parameters (Figure 9), two-way Anova revealed significant effects for the following parameters: main effect of sleep, treatment and interaction in the case of MDA (F = 50.79, η^2^ = 0.54, *p* < 0.0001; F = 11.66, η^2^ = 0.12, *p* = 0.002; F = 6.32, η^2^ = 0.07, *p* = 0.01); main effect of sleep and interaction in the case of GSH (F = 13.89, η^2^ = 0.29, *p* = 0.001 and F = 9.58, η^2^ = 0.20, *p* = 0.004); main effect of treatment, sleep and interaction in the case of GSSG (F = 19.27, η^2^ = 0.25, *p* = 0.0002; F = 25, η^2^ = 0.33, *p* < 0.0001; F = 8.3, η^2^ = 0.11, *p* = 0.0082); main effect of sleep and treatment in regards to GSH/GSSG ratio (F = 16.59, η^2^ = 0.35, *p* = 0.0004 and F = 6.22, η^2^ = 0.13, *p* = 0.019). Compared to control, SD determined a significant increase in MDA and GSSG (*p* < 0.05 and *p* < 0.0001, respectively), coupled with significant decreases in GSH/GSSG ratio and GSH (*p* < 0.01 and *p* < 0.001), pointing towards oxidative stress in the aorta. Furthermore, SD determined a significant increase in GSSG and a significant decrease in the GSH/GSSG ratio in comparison to the CM group (*p* < 0.0001 and *p* < 0.001). Additionally, *C. mas* treatment following SD determined a normalisation of GSSG to levels similar to control (SD vs. SD+CM, *p* < 0.001 and SD+CM vs. control, *p* > 0.05), coupled with a significant increase in lipid peroxidation (*p* < 0.01), possibly suggesting a dual effect of the extract.

Two-way ANOVA revealed a significant main effect of sleep in the case of aortic IL-1β (F = 11.47, η^2^ = 0.40, *p* = 0.0038) and IL-4 (F = 13.09, η^2^ = 0.43, *p* = 0.0023). Post-hoc comparisons revealed a significant increase in IL-4 in the SD group when compared to control (Control vs. SD, *p* < 0.05) and a significant increase in IL-1β levels when compared to the CM group (SD vs. CM, *p* < 0.05), possibly suggesting altered inflammatory pathways in the aorta (Figure 10).

While two-way ANOVA revealed a significant main effect of treatment in the case of testicular GSH (F = 4.78, η^2^ = 0.15, *p* = 0.03), a significant main effect of sleep in regards to GSSG (F = 4.46, η^2^ = 0.15, *p* = 0.045) and a significant interaction effect between sleep and treatment in the case of CAT (F = 5.11, η^2^ = 0.15, *p* = 0.03), these changes failed to reach statistical significance in post-hoc tests (Figure 11).

In the case of testicular cytokines, two-way ANOVA highlighted a significant main effect of sleep in the case of testicular IL-1β (F = 35.64, η^2^ = 0.68, *p* < 0.0001). Post-hoc comparisons revealed a significant increase in IL-1β levels following SD compared to both Control and CM (*p* < 0.01), pointing towards a proinflammatory state in this tissue. While *C. mas* administration decreased IL-4 levels in comparison to SD alone, these changes did not reach statistical significance. Results regarding testicular cytokines are presented in Figure 12.

## 4. Discussion

This study assessed oxidative, inflammatory, and ultrastructural responses across multiple body sites following seven days of REM SD in rats. SD induced redox imbalance and ultrastructural injury in the liver, as well as changes indicative of oxidative stress in the kidneys, spleen, and aorta; these changes were accompanied by organ-specific cytokine increases (hepatic IL-6, testicular IL-1β, aortic IL-4). *C. mas* extract showed limited hepatoprotective effects (ultrastructural and a decrease in IL-6), but presented dual action in the aorta, both as a mild antioxidant (decreased GSSG, *p* < 0.001) and as a pro-oxidant in regards to lipid peroxidation (increased MDA, *p* < 0.01). To our knowledge, this is the first study that evaluated the peripheral effects of cornelian cherry in an SD paradigm and highlighted an in vivo potential pro-oxidant effect.

Globally, these findings align with two recent systematic reviews [9,10], which demonstrate that the oxidative stress induced by SD extends beyond the brain. Specifically, multiple other studies have shown changes in oxidative stress parameters mainly in the liver [33,34,35,36,37], but also in the spleen [38,39] and aorta [16,40,41] in rodent models of PSD or TSD of varying lengths. However, it is worth noting that contrasting results are also available [42,43,44,45,46,47], most likely due to an inherent variability in SD protocols, durations, and possible species or strain differences in the animal models used [9,10].

Physiological sleep regulation is realised by a complex interplay in which several cytokines (e.g., IL-1 and TNF-α, among others) are known to play essential roles (reviewed in [48,49]). However, several datapoints seem to suggest a bidirectionality in the relationship between sleep and cytokine-driven inflammation. In preclinical models, sleep deprivation or fragmentation (SF) has been shown to induce a systemic inflammatory phenotype, characterized by increased gene expression and protein levels of multiple pro-inflammatory cytokines in both central and peripheral tissues (e.g., brain areas—[50,51,52]; peripheral sites—liver [28,53,54], spleen [53], testicle [55]), possibly even extending towards a cytokine storm syndrome [56]. The clinical translation of inflammation following SD remains largely inconclusive, as human studies have reported conflicting findings (e.g., [48,57]).

Our findings support the notion that SD induces a feed-forward loop of oxidative stress and inflammation in the liver that culminates in hepatocellular injury; similar findings regarding the liver response to SD/SF are well known in animal models (e.g., oxidative dysregulation [33,34,35,36,37] and inflammation [28,53,54]). Interestingly, SD has also been shown to increase liver susceptibility to future oxidative insults [58]. Liver ultrastructure changes, such as hepatocyte membrane damage, have also been previously shown following SD. In addition, both liver and metabolic deficiencies have been highlighted in animal studies following SD (e.g., increased liver enzymes, decreased liver protein synthesis, hyperglycaemia, and hyperinsulinemia) [37]. Collectively, these alterations provide a mechanistic framework that helps explain the pathological associations between short sleep and several adverse health outcomes observed in humans, such as metabolic dysfunction-associated fatty liver disease (MAFLD) [59] or hepatocellular carcinoma risk and chronic liver disease mortality [60].

Scarce data are available regarding kidney oxidative stress after SD. While some data may suggest a greater resilience of renal tissue to oxidative stress [43,47], other data points towards redox imbalance only in conjunction with chronic intermittent hypoxia [45]. However, several studies have reported histological alterations in this organ [61], altered metabolite profiles [62], and increased blood urea nitrogen indicative of kidney injury [42] in the context of SD. Although not confirmed, the oxidative challenge induced by SD has been hypothesised to be a contributing factor to these changes [61]. Furthermore, SD during pregnancy seems to induce long-lasting kidney abnormalities in offspring [63]. Taken together, these alterations might provide a conceptual framework for further studies to explore the known U-shaped association between sleep duration and kidney disease in humans [64].

Conflicting data are available in the case of the spleen response to SD. While some studies have highlighted an oxidative challenge following SD [38,39], others failed to report this [38,46]. However, the spleen may respond to SD through other mechanisms such as increased nitrotyrosine, large subunit caspase-3, and modest white pulp cell proliferation [46] or through altered B lymphopoiesis [39].

We found no significant intergroup differences in testicular oxidative stress parameters, despite a significant increase in IL-1β due to SD. These findings contrast with prior reports of testicular sensitivity to SD-induced oxidative stress [55,65]. Furthermore, reports on testicular cytokines following SD present mixed results [55,65]. Although ROS-driven NLRP3 activation is known as a classic trigger of IL-1β release, ROS-independent pathways have also been proposed (reviewed in [66]). Sertoli cells have been shown to generate IL-1β via NLRP3 inflammasome activation [67], and NLRP3 activation has been linked to male infertility in both preclinical and clinical studies [68], providing a potential mechanistic pathway that connects SD to impaired sperm parameters in humans [69].

In the aorta, SD influenced most examined oxidative parameters (significant increase in MDA—*p* < 0.05, GSSG—*p* < 0.0001; significant decrease in GSH—*p* < 0.001, GSH/GSSG ratio—*p* < 0.01); furthermore, SD determined a concomitant increase in IL-1β (*p* > 0.05) and a significant increase in IL-4 (*p* < 0.05), possibly pointing towards a compensatory mechanism [70]. Aortic lipid peroxidation has been previously highlighted following both shorter and longer SD paradigms [16,40], coupled with decreased ARE and PON enzyme activities, possibly suggestive of endothelial dysfunction [16]. Decreased aortic nitric oxide has also been reported following SD [16,41]. Notably, the vascular effects of SD appear to be age-dependent, with increased blood pressure and impaired aortic relaxation observed in middle-aged but not younger rodents, likely mediated through alterations in the eNOS/NO/cGMP pathway [41]. Functionally, these changes seem to provide mechanistic links for the known impact of short sleep on several CVD pathologies [1].

Consistent with our previous report aimed at central sites [26], *C. mas* extract exerted overall modest modulatory effects. These effects were most evident in the aorta and, to a lesser extent, in hepatic ultrastructure and IL-6 levels. In the aorta (Figure 9D), the extract normalized GSSG levels previously increased by SD (SD vs. SD+CM, *p* < 0.001), indicating some antioxidant activity; however, it concurrently elevated MDA (SD vs. SD+CM, *p* < 0.01) (Figure 9A), possibly pointing towards dysregulated redox balance with enhanced lipid peroxidation and a pro-oxidant effect. The effect of exogenous antioxidants is often pleiotropic (either anti- or pro-oxidant), determined by various factors ranging from the specific antioxidant to circadian influence and others (reviewed in [24,71]). Globally, polyphenols are known to be characterised by low bioavailability, rapid metabolism, and complex in vivo pharmacokinetics [24].

Various *C. mas* extracts have been used before in preclinical models, thus suggesting beneficial antioxidant or anti-inflammatory effects at different doses [72,73,74,75]. The effects of *C. mas* extracts have also been investigated in clinical trials (e.g., [76]), without a focus on their antioxidant properties. As far as we are aware, reports of pro-oxidant activity of *C. mas* are limited to two in-vitro studies employing an ethanolic fruit extract and an aqueous extract of leaves; both studies tentatively point toward a dose-dependent response, with higher doses having a pro-oxidant effect [77,78].

The dose of cherry extract used in this study (30 mg polyphenols/kg bw/day) exceeds that reported in other rodent studies (e.g., [79]); however, far higher doses of polyphenols (800–1000 mg/kg bw/day) seem well tolerated in prolonged administration [80,81]. Polyphenol pharmacokinetics can vary widely [24], with compounds such as quercetin showing site-specific distribution in rodents [82]. An increased uptake of bioactive compounds (either polyphenols or secondary metabolites) in the aorta as compared to other body sites might partially explain our findings.

The physiological context of SD regarding microbiome alterations might at least partially explain the observed results. SD-induced gut dysbiosis (reviewed in [83,84,85]), mediated through mechanisms such as ROS accumulation in the gut [86], may represent a mechanistic link between altered sleep health and cardiovascular disease [84,87]. Furthermore, the gut microbiome seemingly has a complex relationship with dietary antioxidants. Moderate blueberry polyphenol intake appears to enhance microbiome diversity in rats, whereas higher doses may reduce it [88], potentially linking SD, microbiome disruption, dietary antioxidant intake, and the observed increase in aortic lipid peroxidation.

Prolonged SD inherently involves circadian disruption [89]. Furthermore, preliminary data seem to suggest a circadian influence on the response to exogenous antioxidants in both rodents [90] and humans [91]. Resveratrol seems to exhibit pro-oxidant effects during the rodents’ rest phase and antioxidant effects during the active phase [90]. Bioactive compounds in *C. mas* may show a similar pattern, though further confirmation is needed.

The time relationship to the oxidative challenge warrants exploration in further studies. In this study, *C. mas* was administered for seven days during the duration of the SD protocol. Previous studies have demonstrated potential antioxidant benefits of pre-treatment [14] and the combination of pre-treatment with concomitant treatment [15,92] using various compounds in rodent SD protocols. Multiple lines of evidence seem to support the view that the mechanism of action for various natural antioxidants is more accurately explained by their capacity to stimulate cellular redox activity, rather than inhibit it as previously believed (reviewed in [24]). While further confirmation is required, it is possible that treatment during the oxidative challenge of SD might overwhelm the cellular redox defences, ultimately leading to lipid peroxidation. Furthermore, pre-treatment may potentially prime the cellular antioxidant defences for a future oxidative challenge.

Although the MMSP paradigm is the most commonly employed REM SD protocol in rats [9], previous studies have shown that it may also influence slow-wave sleep (SWS) [27]. In comparison to other studies, we employed a single cage control group in this study. While the lack of a large platform group may be seen as a limitation of the study, previous findings seem to suggest that redox parameters show only minimal differences between large-platform and cage-control groups [9]. Furthermore, large platforms may also significantly reduce both REM and NREM sleep [27], may be similarly stressful [93], potentially making them unsuitable as a control group.

To the best of our knowledge, this study represents the first experimental investigation into the potential modulatory action of a *C. mas* extract on peripheral organs in the context of PSD. However, several limitations of this study should be acknowledged, including the exclusive use of adult male Wistar rats, the application of a single SD protocol in terms of both duration and type, and the administration of a single dose of the plant extract during the deprivation period. A further limitation is the lack of semi-quantitative TEM analysis, which may have provided additional insights. Additionally, gene expression of antioxidant enzymes and cytokines was not evaluated in this study. The potential for clinical translation represents both an important limitation of the current study and an important focus for future investigation, with a current paucity of data regarding the modulatory action of natural extracts in the context of SD in human models.

Taken together, our results seem to point towards a dual effect of the *C. mas* extract in the aorta of REM SD Wistar rats. However, the pro-oxidant effect of this extract is based solely on aortic lipid peroxidation. While based on limited data, the potential pro-oxidant effects of this extract may be best explained through the interplay between prolonged SD and the bioactive compounds in *C. mas*.

## 5. Conclusions

Based on our previous findings indicating overall modest central effects (evidenced mainly on behavioural and morphological parameters), along with the current results showing primarily increased lipid peroxidation in the aorta and minimal impact on other organs, it appears that *Cornus mas* fruit extract is unlikely to provide significant benefits in the context of prolonged REM SD in Wistar rats. However, different extract types, dosages, or REM sleep deprivation durations may potentially lead to different outcomes. To our knowledge, this is the first study to examine the effects of *Cornus mas* on multiple peripheral body sites in rodents under sleep deprivation, highlighting the potential dual effects of this natural extract in this context.

These results further highlight the potential dual effects of natural extracts. However, it should be noted that the presumed pro-oxidant effect of *Cornus mas* is inferred in this study solely from increased lipid peroxidation in the aorta. Confirmation of these effects, followed by the evaluation of the underlying molecular mechanisms and pathways, warrants further exploration in future studies, as these mechanisms may also extend to other natural extracts in the context of sleep deprivation.

## Data Availability

Data are included in the Manuscript and further available from the corresponding author upon reasonable request.
